# Global Analysis of Membrane-associated Protein Oligomerization Using Protein Correlation Profiling*[Fn FN2]

**DOI:** 10.1074/mcp.RA117.000276

**Published:** 2017-09-08

**Authors:** Zachary McBride, Donglai Chen, Christy Reick, Jun Xie, Daniel B. Szymanski

**Affiliations:** ‡Department of Biological Sciences, Purdue University, West Lafayette, Indiana;; §Department of Statistics, Purdue University, West Lafayette, Indiana;; ¶College of Osteopathic Medicine, Marian University, Indianapolis;; ‖Department of Botany and Plant Pathology, Purdue University, West Lafayette, Indiana

## Abstract

Membrane-associated proteins are required for essential processes like transport, organelle biogenesis, and signaling. Many are expected to function as part of an oligomeric protein complex. However, membrane-associated proteins are challenging to work with, and large-scale data sets on the oligomerization state of this important class of proteins is missing. Here we combined cell fractionation of Arabidopsis leaves with nondenaturing detergent solubilization and LC/MS-based profiling of size exclusion chromatography fractions to measure the apparent masses of >1350 membrane-associated proteins. Our method identified proteins from all of the major organelles, with more than 50% of them predicted to be part of a stable complex. The plasma membrane was the most highly enriched in large protein complexes compared with other organelles. Hundreds of novel protein complexes were identified. Over 150 proteins had a complicated localization pattern, and were clearly partitioned between cytosolic and membrane-associated pools. A subset of these dual localized proteins had oligomerization states that differed based on localization. Our data set is an important resource for the community that includes new functionally relevant data for membrane-localized protein complexes that could not be predicted based on sequence alone. Our method enables the analysis of protein complex localization and dynamics, and is a first step in the development of a method in which LC/MS profile data can be used to predict the composition of membrane-associated protein complexes.

Plants provide food, fiber, and a rapidly growing list of renewable bioproducts that supply the energy, pharmaceutical, and chemical industries. In response to increased demand from a growing global population and climate instability, the next generation of crops will require rational design and engineering where plant architectures are optimized for specialized environments and metabolism is altered to generate products with increased value (1). This is a tall order to fill, and success on this front will require broad systems-level knowledge about cell function and how proteins, protein complexes, and networks of interacting pathways determine plant traits (2). Modern genetic approaches provide a reliable gateway into cellular control, and are increasingly efficient in terms of discovering the composition and regulation of protein complexes that dictate phenotypes. However, there are likely to be thousands of protein complexes in the cell that remain unknown (3). The analytical power of protein mass spectrometry presents an opportunity for efficient protein complex discovery, and this article describes new methods for the high throughput analysis of membrane-associated proteins that have previously escaped functional analyses.

Membrane-associated proteins are important because subcellular compartmentalization is a defining feature of eukaryotic cells. Highly specialized protein complexes assemble on or within organelles to compartmentalize gene expression, carry out electron transport (4, 5), generate unique pH environments (6), and to customize metabolism for key activities such as cell wall polysaccharide synthesis (7). Organelle functions are interdependent, and protein complexes often reside at multiple locations to enable vesicle trafficking (8) and promote efficient exchange of metabolic intermediates (9, 10). During signaling, protein complexes are deployed at the plasma membrane (11–16) or the endoplasmic reticulum (ER) surface (13, 17) to sense hormones, pathogens, light quality and transmit information to other cellular locales. Proteins and protein complexes often have multiple localizations within a single cell (18). This can reflect active and inactive pools (19), distinct functions at different organelles, or similar functions at multiple locations (20, 21).

There are dozens of high throughput methods to detect protein-protein interactions. However, most are designed for soluble proteins and few retain information about cellular localization. A common method such as the yeast 2-hybrid assay has been adapted to detect physical interactions among membrane-associated proteins (21). Tandem affinity purification (TAP) coupled with LC/MS/MS is another powerful high-throughput method to discover protein complexes (22), and has revealed novel membrane associated protein complexes (23). The disadvantage of TAP is that each test protein must be cloned, transformed into a cell line or intact organism, and expressed in a functional state at native levels.

Protein mass spectrometry and protein correlation profiling (PCP) is an emerging technique to analyze endogenous protein complexes as they are partially purified under native conditions. In this technique the relative abundance of hundreds to thousands of endogenous proteins are quantified across the fractions following some kind of separation strategy. The strength of PCP is that thousands of proteins are analyzed in a single experiment for unbiased discovery of stable protein complexes. One limitation this method is that elongated protein shapes or high-mass post-translational modifications can lead to false positives. This approach was first used with native gels and sizing columns to discover large complexes in the chloroplast stroma (24, 25) and subsequently protein complexes from microbes, plants, and vertebrate samples have been analyzed in this manner (25–30). Improvements in LC/MS/MS instrumentation have allowed several groups to use combinations of profile data and bioinformatics to predict protein complex composition (28, 31, 32). The above profiling articles analyze soluble proteins, and the important membrane-associated proteins were discarded as the troublesome microsomal pellet.

This article describes new methods to analyze the oligomerization state of membrane-associated complexes isolated from Arabidopsis leaves. This cellular fraction is recalcitrant to analysis because microsomal proteins tend to be hydrophobic and/or strongly associated with membranes, and nondenaturing detergents are needed to efficiently solubilize yet retain protein-protein interactions. Previously, microsomal proteins complexes have been profiled by purifying an organelle, solubilization of proteins with a nondenaturing detergent, followed by density gradient centrifugation (33) or nondenaturing gel electrophoresis (34). In a recent publication, chemical crosslinking was used to covalently link neighboring proteins so that size fractionation and protein correlation profiling could be conducted under denaturing conditions (35). Although the procedure precludes accurate apparent mass determinations using SEC and nonspecific cross-linking led to some peak broadening and increased baseline signals, membrane-associated complexes were clearly identified in that study. Here we use the nondenaturing ionic detergent cholate and SEC to analyze the oligomerization state of membrane-associated proteins. Reproducible protein profiles with high dynamic range were generated using label free LC/MS/MS and extracted ion chromatograms (XIC)^1^-based quantification. Peak detection algorithms were developed to deconvolute complex profiles into one or more resolvable peaks, and allowed us to predict hundreds of protein complexes with diverse subcellular localizations and functionalities. This new analytical pipeline enables large-scale functional analyses of protein complexes that were previously intractable.

## EXPERIMENTAL PROCEDURES

### 

#### 

##### Microsome Isolation

Plants were grown and 2 grams of leaf tissue was harvested as described by Aryal *et al.*, (2014) (29). Leaves were homogenized using a polytron (Brinkman Instruments, Hampton, NH) in microsome isolation buffer (MIB) buffer (50 mm HEPES-KOH pH 7.5, 250 mm sorbitol, 50 mm KOAc, 2 mm Mg(OAc)_2_, 1 mm EDTA, 1 mm EGTA, 1 mm DTT, 2 mm phenyl methyl sulfonylfluoride and 1% (v/v) inhibitor mixture (160 mg/ml benzamidine-HCl, 12 mg/ml phenanthroline, 0.1 mg/ml aprotinin, 100 mg/ml leupeptin, and 0.1 mg/ml pepstatin A). Debris was cleared by four layers of cheesecloth and centrifugation at 1 k x g (Beckman Avanti 30, Indianapolis, IN) for 10 min, 4 °C. The microsomal fraction (P200) was enriched by ultra-centrifugation of the 1 K supernatant at 200 k x g for 20 min, 4 °C (Beckman Optima Ultracentrifuge). Weakly associated soluble proteins were depleted by two washes with 12 ml of MIB. Sodium cholate, Triton-x 100, sodium deoxycholate, and dodecyl-β-d-maltoside were screened by protein concentration and Western blot for their ability to solubilize microsomal proteins and found to be less efficient than cholate. The enriched membranes were solubilized by cholate as described by Basu *et al.*, (2008) (36). Briefly, the microsomal fraction was solubilized in 4% cholate at a protein concentration of 2.5 μg/μl for 1 h at room temperature and solubilized proteins were isolated by ultra-centrifugation.

##### Size Exclusion Chromatography

Protein separation was performed by size exclusion chromatography on an AKTA FPLC system (GE Life sciences, Pittsburgh, PA) with a Superose 6 10 × 300 GL (GE Healthcare) or a Superdex 200 10/300 GL (GE Healthcare) column at 6 °C. The mobile phase (50 mm HEPES-KOH pH 7.8, 100 mm NaCl, 10 mm MgCl_2_, 5% glycerol and 0.1% (w/v) sodium cholate) was passed over the column at a flow rate of 0.4 ml/min while the elution profile was monitored by absorbance measured at 280 nm. The SEC column was calibrated with the gel filtration kit 1000 (MWGF1000, Sigma-Aldrich, St. Louis, MO) with a mass range from 669 kDa to 29 kDa to determine a protein's apparent mass. Twenty-four 500 μl fractions were collected, starting one fraction prior to the void measured by blue dextran.

##### Gel Electrophoresis, Staining, and Protein Immunoblot Analysis

Proteins from the SEC and sucrose velocity gradients were visualized by SDS-PAGE. Protein samples at equal proportions were resuspended in Laemmli buffer (0.1 M Tris-HCl, pH 6.8, 1% SDS, 5% glycerol, and 0.01% bromphenol blue), separated on a 10% SDS-PAGE gel in a Tris-Glycine buffer, and visualized by either silver staining Vorum protocol (37) or Coomassie blue.

Immunoblot analysis was performed as described by Aryal *et al.*, (2014) (29) except for SPK1 (36). SPK1 was resolved on a 7.5% SDS-PAGE gel and the primary antibody was diluted 1 to 2000.

##### Sucrose Velocity Gradient Centrifugation

A 5 ml linear sucrose velocity gradient was created as described by Zhang *et al.*, (2010) (68). The sucrose solution contained 20 mm HEPES-KOH pH 7.5, 2 mm EDTA, 1 mm DTT, 1 mm PMSF, protease inhibitors. A linear 22 to 52% sucrose gradient was formed using a gradient master 108 (BioComp, Fredericton, NB, Canada). Two hundred microliters of the washed P200 pellet was suspended (10 mm HEPES/KOH pH 7.2, 150 mm NaCl, 1 mm EDTA, 10% glycerol, 1% protease inhibitors, 1 mm PMSF) and layered on top of the sucrose gradient. The gradient was centrifuged for 18 h at 100,000 × *g*, 4 °C in a MLS 50 swinging buck rotor. Twenty-five 200 μl fractions were analyzed.

##### Experimental Design and Statistical Rationale

For LC-MS/MS profiling two biological replicates were chosen for the Superdex, Superose, and sucrose velocity gradient based on the high level of reproducibility in between biological replicates from Aryal *et al.*, (2014) (29). Forty-eight Superdex, 24 Superose, and 50 Sucrose velocity gradient fractions were analyzed by LC/MS/MS. For CoIP-MS pull downs three biological replicates were performed with both antibodies against the protein of interest and preimmune sera. In all experiments, the fractions were analyzed on the mass spectrometer by equal proportions. The MaxQuant (38) search was performed with biological replicates searched simultaneously, with alignment between runs to allow for consistent identification and quantification of protein isoforms. A two-fraction or less shift in the chromatography fractions was used as a criterion to accept reliable peaks in the profile data between biological replicates of a given separation approach.

##### LC/MS Sample Preparation and Analysis

LC-MS/MS was performed on peptides from SEC and sucrose velocity gradient fractions. Buffer was removed and proteins were concentrated by precipitation with 4 volumes of ice cold 100% acetone, stored overnight at −20 °C and pelleted by centrifugation at 15,000 × *g* for 20 min at 4 °C. The protein pellet was washed 2× with 300 μl ice cold 80% acetone and dried. Proteins were suspended in 8 m urea, then reduced by 10 mm DTT at 60 °C for 45 min and alkylated with 20 mm iodoacetamide in the dark at room temperature. Urea was diluted to 1.5 m with 50 mm ammonium bicarbonate, and trypsin digestion was done using porcine trypsin (Sigma-Aldrich) at an enzyme to protein ratio of 1 to 50 at 37 °C. After 5 h trypsin was resupplied in at 1:100 and proceeded overnight. Residual detergent in SEC experiments was depleted with 0.5 ml detergent removal columns (Pierce Biotechnology, Rockford, IL). Peptides were desalted by Pierce C18 spin columns (Pierce Biotechnology), dried, and suspended in 3% acetonitrile, 0.1% FA. All samples from a biological replicate were adjusted to the same volume, so that the most concentrated sample had a concentration of 0.3 μg/μl.

##### LC-MS/MS AB Sciex 5600

SEC samples were analyzed by LC-MS/MS as described by Aryal *et al.*, 2017 (55). Briefly, using an Eksigent nano-LC 425 HPLC (Dublin, CA), 5 μl of peptides were resolved over a 90 min gradient from 0 to 35% acetonitrile in 0.1% FA prior to analysis on an AB Sciex quadrupole time-of-flight (QqTOF) TripleTOF 5600, (Framingham, MA). Data was collected in a data-dependent mode with the 50 most abundant precursor ions selected for MS/MS.

##### LC-MS/MS Thermo Fisher Q-Exactive

Sucrose velocity gradient fractions were analyzed by LC-MS/MS on a Thermo Scientific Q Exactive Orbitrap Mass spectrometer in conjunction with Proxeon Easy-nLC II HPLC (Thermo Fisher Scientific, Waltham, MA) and Proxeon nanospray source. Peptides were loaded on a 100 micron × 25 mm Magic C18 100Å 5U reverse phase trap prior to a 75 micron × 150 mm Magic C18 200Å 3U reverse phase analytical column. Peptides were eluted over a 125 min gradient with a flow rate of 300 nL/min. An MS survey scan was obtained from 350–1600 *m*/*z*, and MS/MS spectra were acquired by selecting the 15 most abundant precursor ions for sequencing with high-energy collisional dissociation normalized collision energy of 27%. To reduce the number of times the same ion was sequenced, a fifteen-second dynamic exclusion window was used.

##### Peptide Identification and Quantification

The MaxQuant software package (v. 1.5.3.28) (38) searched the .wiff and corresponding .wiff.scan or .raw files to identify and quantify peptides. The MaxQuant search was performed using the TAIR (The Arabidopsis Information Resource) protein sequence database version 10 (TAIR10; 35386 protein sequences, 14,482,855 residues). The parameters were set as follows; enzyme specificity was trypsin, with up to two missed cleavages; Carbamidomethyl of cysteine was a fixed modification, acetyl (protein N-term), and oxidation of methionine were variable modifications. The mass tolerance for the precursor ions was 10 ppm and the fragment ions were set to 40 ppm. The protein and peptide false discovery rate was limited to 1% with a reverse decoy database. Proteins identified by a single peptide were accepted when the score was ≥ 5. Peptides abundance was calculated from the extracted ion current for both razor (shared peptides that can be assigned to a protein based on unique peptides and the principle of Occam's razor) and unique peptides, and an aggregate peptide intensity was used to calculate protein level signals. The raw XIC based intensity values were used to determine the peaks in a protein elution profile because all fractions were loaded onto the MS by equal proportions. Protein groups and peptide tables were exported as .txt files to Microsoft Access and Excel where subsequent data analyses were performed. For functional analysis, the predominant protein group was used to cross reference functional information from the SUBAcon (39), MAPMAN (40), Uniprot (uniprot.org) or TAIR (Arabidopsis.org) databases.

##### Gaussian Peak Fitting, Reproducibility, and R_app_ Calculations

A Gaussian fitting algorithm adapted from Kristensen *et al.*, (2012) (41) was optimized to fit the resolution of the separation technique and reduce over fitting. To fit our profiling experiments, we required each protein be identified in three fractions with two being adjacent. Each peak must be separated by ≥4 fractions, and additional peaks must have an intensity ≥20% of the most intense peak. To prevent over fitting, a Bayesian information criterion (BIC) was used to calculate the optimal number of peaks, calculating the goodness of fit of the fitted peak to the raw data and penalizing for each additional peak that was added (42). The number of Gaussian peaks fitted to the data was determined by the lowest BIC. A protein was determined reproducible between the two biological replicates if the fitted peak from the two replicates had ≤2 fraction shift. If a protein profile was not fitted to a Gaussian curve, the global max was used. All nonreproducible peaks were omitted from further analysis.

From the peak(s) the apparent mass of the proteins was estimated. First, peaks were defined as unresolved if they were not at least one fraction outside of the void. From the resolved peaks, the M_app_ of the protein was calculated using the regression line from SEC standards. The oligomerization state (R_app_) was predicted by the ratio of the M_app_ to the monomer mass of the protein calculated based on the TAIR proteome annotation. A protein was predicted as a complex if the peak was reproducible (less than a two-fraction shift between biological replicates) and the R_app_ was ≥2 in one biological replicate.

##### Prediction of Orthologous Protein Complexes Using Known Metazoan Complexes

The comprehensive resource of mammalian protein complexes (CORUM) (43) (11/2015), a database of known metazoan protein complexes, was downloaded. Arabidopsis orthologs were identified by Metaphors (44) and inParanoid (45), and then matched to metazoan complexes. Redundancies were removed when the same complex was identified across multiple metazoan species. Coverage of the predicted complexes in Arabidopsis was defined as high, medium, or low based on the following criteria: high coverage (orthologs identified for ≥80% subunits, 3 of 4 subunits, or 2 of 3 subunits); medium coverage (60% ≤ orthologs ID < 80%); and low coverage (40% ≤ orthologs ID < 60%). The M_calc_ of the assembled metazoan complex was calculated by summing the M_mono_ of all subunits. The M_calc_ of predicted Arabidopsis complexes was calculated when there was 100% ortholog coverage.

##### Coimmunoprecipitation Mass Spectrometry

Coimmunoprecipitation was performed as described by Aryal *et al.*, 2017 (55) for membrane-associated NITRILASE1. Briefly, leaves were frozen in liquid nitrogen, homogenized, and differential centrifugation enriched microsomal proteins. Microsomal protein was solubilized with sodium cholate as described above. Mass spectrometry analysis was performed on the Q Exactive mass spectrometer. Protein hits were filtered to require each protein to be identified with two or more peptides. Positive CoIP interactors were defined as being identified in two of three biological replicates containing the antibody of interest and zero intensity in the negative controls or three biological replicates containing the antibody of interest and in one or zero of the negative controls.

## RESULTS

### 

#### 

##### Workflow: Identification of Microsomal Protein Oligomerization State

**Fig. 1.**
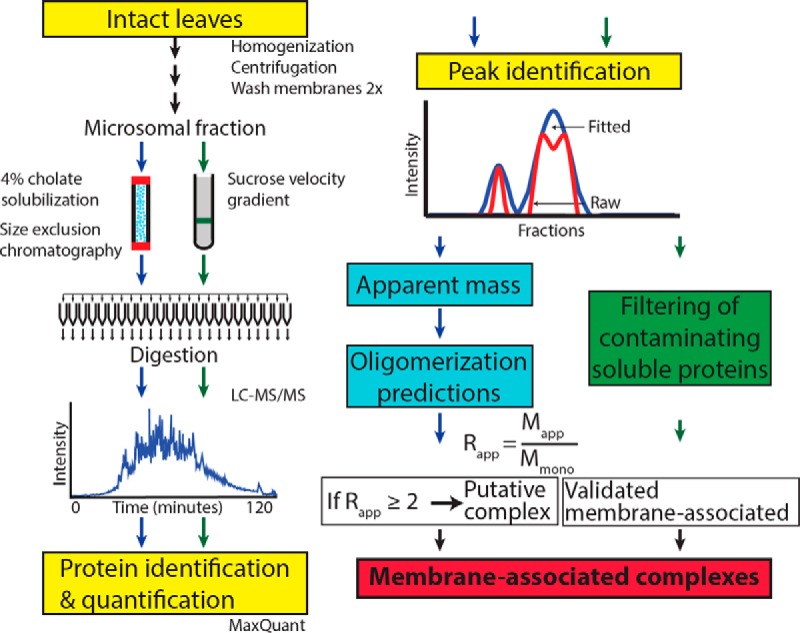
**Workflow for a proteomic analysis of membrane-associated protein complexes.** Arabidopsis leaves were homogenized and a crude microsomal fraction was isolated. Microsomal proteins were solubilized in cholate and resolved by size exclusion chromatography. Fractions were collected, digested, and analyzed by LC-MS/MS. MaxQuant was used to identify the peptides and generate XIC abundance profiles. Peaks in the elution profiles were identified using Gaussian fitting. From the SEC profiles protein oligomerization predictions were made by calculating the ratio (R_app_) of the apparent mass (M_app_) determined by SEC to the monomer mass (M_mono_) of the protein. Proteins with a R_app_ ≥2 were predicted to form a putative complex. Contaminating soluble proteins were removed from the data set based their inability to sufficiently penetrate a sucrose gradient following ultracentrifugation (green box).

The objective of this work is to develop an analytical pipeline to analyze the oligomerization state of membrane-associated proteins. Our approach measures the apparent mass of native membrane-associated protein complexes that are solubilized from washed microsomes using label-free quantitative proteomics (Fig. 1). Starting with leaves, the membrane fraction was enriched by homogenization and differential centrifugation. The membrane-associated proteins were solubilized with a nondenaturing detergent and separated using FPLC and a high-resolution SEC. Each SEC fraction was then analyzed by label-free mass spectrometry to identify the proteins and quantify their relative abundance across the fractions. The peak in the protein's abundance was used to calculate the apparent mass (M_app_) of the endogenous protein. Oligomerization predictions were made by the calculated ratio (R_app_) of the M_app_ to the monomer mass calculated from the primary amino acid sequence (M_mono_). When the R_app_ was ≥ 2 the protein was predicted to form a complex. Profiling of a sucrose velocity gradient of microsomal membranes was used to determine which of proteins identified in both the cholate-soluble fraction and cytosol fraction were “true” membrane-associated *versus* cytosolic contaminants based on penetration depth into the sucrose gradient. This profiling approach allows for the oligomerization state of thousands of proteins to be monitored in a single experiment, an important step toward gaining systems levels knowledge about protein complexes in the endomembrane system.

##### Solubilization of Microsomal Proteins

**Fig. 2.**
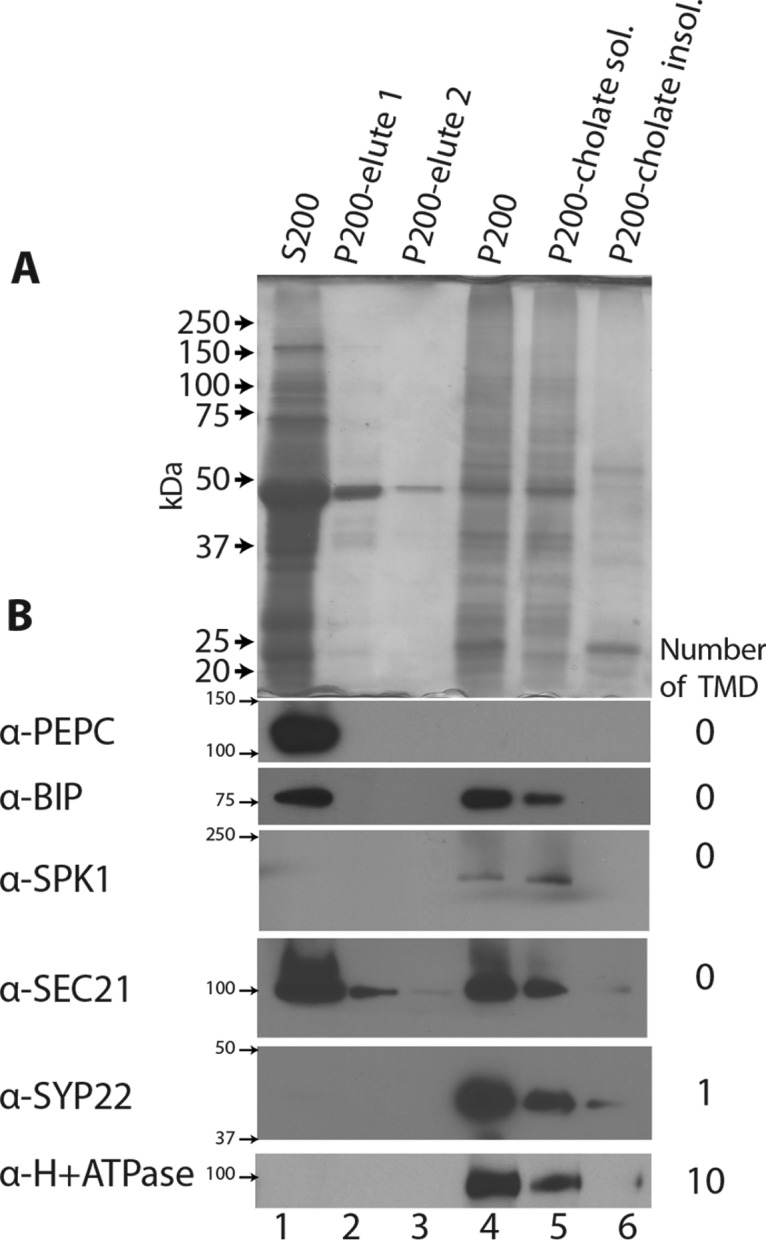
**Cholate effectively solubilizes microsomal proteins.** *A*, The soluble and membrane fractions were obtained by differential centrifugation, resolved on a SDS-PAGE gel by equal proportions and visualized by staining. S200 represents soluble proteins in the crude cytosol fraction. P200-elute 1 and p200-elute 2 were the supernatants from the respective washes. The microsomal fraction (P200) was resuspended in 4% cholate to solubilize membrane-associated proteins. After ultra-centrifugation the cholate-solubilized proteins were in the supernatant (P200-Cholate Sol.) and the insoluble proteins in the pellet (P200-cholate insol.). *B*, Western blot analysis of cholate solubilization of a panel of proteins with differing solubilities. Cytosolic PHOSPHOENOL-PYRUVATE CARBOXYLASE (PEPC), BINDING IMMUNOGLOBULIN PROTEIN (BIP), SEC21, and SPIKE1 (SPK1) (36) represent proteins that do not contain a transmembrane domain; SYP22 (52) has a single transmembrane domain, and H+ ATPase contains multiple transmembrane domains.

To deplete weakly associated, contaminating proteins from the microsomes, membranes were washed twice. An SDS-PAGE gel of the washed fractions indicated many weakly-associated proteins were eluted, including a prominent ∼50 kDa band that corresponded to the large subunit of ribulose-1,5-bisphosphate carboxylase/oxygenase (RUBISCO), the most abundant protein in plant cells (Fig. 2*A*, lanes 2 and 3). Depletion of highly abundant proteins like RUBISCO is required because during mass spectrometry it can lead to ion suppression and artifactual peaks in the elution profiles (29). Approximately 30% of the total protein was weakly associated with membranes, but even after repeated washes weakly associated proteins such as RUBISCO were present in the P200 fraction (Fig. 2*A*, Lane 4). In this study, the washed P200 was defined as the enriched microsomal fraction and true membrane-associated proteins will be identified based on sedimentation with a sucrose velocity gradient (see below). Not all soluble proteins are contaminating the P200. PEPC, a known cytosolic enzyme localized exclusively to the soluble fraction (Fig. 2*B*).

The nondenaturing detergent sodium cholate was chosen based on its ability to solubilize active enzymes (46, 47), protein complexes for crystallization (48), and intact membrane-associated proteins under native conditions (36, 49). Cholate solubilization was efficient based on a Bradford assay, with ∼70% of the microsomal proteins partitioned into the soluble phase. Solubilization efficiency of selected proteins with differing types of membrane association in the cell were tested by Western blot (Fig. 2*B*). Cholate solubilized the endoplasmic reticulum membrane and released the luminal protein BIP (50). The two peripheral membrane proteins, the guanine nucleotide exchange factor SPIKE1 (SPK1) (36) and the COPI coat protein secretory 21 (SEC21) (51) were almost completely solubilized. Proteins containing either a single (syntaxin 22 (SYP22) a t-SNARE (52)) or multiple transmembrane domains (the plasma membrane proton ATPase (53) were both efficiently solubilized, indicating that cholate has the ability to solubilize a wide range of membrane-associated proteins.

##### Microsomal Profiling Provides Functional Characterization for Thousands of Proteins

SEC was used for large-scale apparent mass determinations for the cholate-soluble proteins. The absorbance profile from the Superdex 200 10 × 300 GL column indicated that many of the proteins eluted as large complexes or aggregates in the void; however, most of the protein eluted within the resolving range of the column (supplemental Fig. S1*A*). A silver stained SDS-PAGE gel showed qualitative evidence for oligomerization; for example, the proteins in fraction 8 had an apparent mass of 180 kDa and multiple proteins were identified with a M_mono_ of <50 kDa, a 5-fraction shift from the expected mass of the monomer (supplemental Fig. S1*B*). A Superose 6 10 × 300 GL with an extended apparent mass resolution up to 5 MDa was used to detect larger complexes. The Superose column resolved many large complexes in the 5 MDa to 5 kDa range, but there was still a large peak in the void, indicating that some of the cholate solubilized proteins are either partially solubilized complexes or large insoluble aggregates that cannot be resolved using SEC. Because most of proteins eluted were within the mass range of our columns, we profiled protein abundance across the entire Superdex elution profile and the nonoverlapping higher mass fractions of the Superose column that spanned a from 5 MDa to 500 kDa using LC/MS/MS and the AB Sciex 5600 mass spectrometer.

**Fig. 3.**
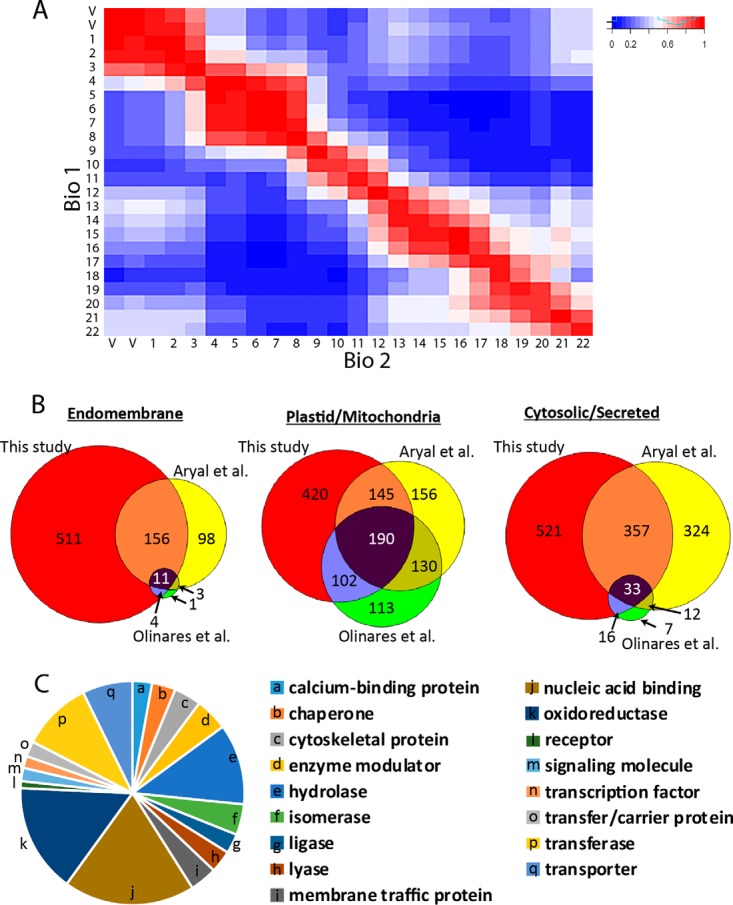
**Reproducible apparent mass determinations for a previously uncharacterized set of microsomal proteins.** *A*, A heat map of the Pearson correlation coefficients of the protein quantification signals in two biological replicates. The matrix was generated by Data Analysis and Extension Tool (DAnTE) (90). *B*, Protein coverage of proteins with a known and single localization based on the SUBAcon database: endomembrane system (Golgi, endoplasmic reticulum, plasma membrane, nucleus, peroxisome), plastid/mitochondria, or cytosolic/secreted. The number of proteins in this study are compared with two others that analyzed soluble proteins in Arabidopsis. *C*, The cholate solubilized fraction includes proteins with diverse functionalities predicted by Panther protein classes (91).

To generate reliable M_app_ measurements two biological replicates were analyzed and only the proteins with reproducible peaks were reported here. MaxQuant was used for protein identification, LC/MS chromatogram alignment, and extracted ion chromatogram (XIC) based quantification (38). The relative abundance of the identified peptides was quantified based on XIC to increase the dynamic range and improve the quantitation of low abundance proteins (54). In addition to XIC quantification, an alignment between runs was performed that allowed peptides to be quantified based on retention time and charge to mass ratio if it was selected for sequencing in a single run (38). MaxQuant identified over ∼13,400 peptides mapping to >2550 proteins at a 1% false discovery rate that were present in both biological replicates (supplemental Fig. S1*C*) (supplemental Table S1). A correlation analysis of the protein intensities among all SEC fractions revealed maximal Pearson correlation coefficients between Bio1 and Bio2 along the diagonal, indicating a consistent identification and quantification of proteins in similar SEC fractions in the two replicates (Superdex: Fig. 3*A*; Superose: supplemental Fig. S1*D*).

The protein identification and oligomerization predictions made in this study were quite distinct from those published previously. For example, we analyzed ∼1450 proteins that have not been characterized in previous work on the oligomerization state of soluble proteins in the cytosol (55) or chloroplast (25) (Fig. 3*B*). Based on the current subcellular localization predictions from SUBAcon (Version: SUBA3) (39), our proteomic data set included many uncharacterized cytosolic, chloroplast, and mitochondrial proteins. The largest enrichment was in endomembrane-localized proteins, with 65% analyzed for the first time. The cholate-soluble fraction in this study also had a higher proportion of proteins predicted with one or more membrane spanning domains that was more representative of the Arabidopsis proteome (supplemental Fig. S1*E*).

##### Gaussian Fitting Identifies Multiple Elution Peaks

**Fig. 4.**
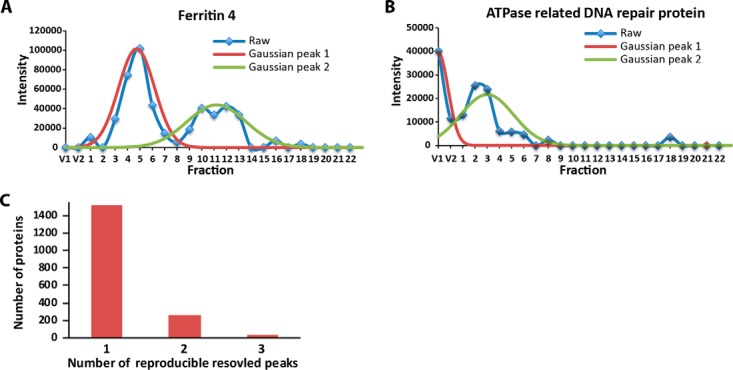
**Gaussian fitting identifies multiple oligomerization states for individual proteins.** *A*, The FERRITIN 4 profile with multiple peaks can be deconvolved into two individual peaks. *B*, Separation of an unresolved peak in the void from a resolved peak for the ATPase-related DNA REPAIR PROTEIN. *C*, The number of protein groups in which 1, 2, or 3 reproducible peaks were identified.

When the protein abundance profiles were plotted, many contained multiple peaks. This is biologically relevant data that can reflect a protein existing in multiple oligomerization states in the cell. To extract this information in an automated manner the profiles were deconvolved into one or more Gaussian peaks (41), and peak locations that were reproducible in the two biological replicates were accepted as reliable. For example, FERRITIN4 a known homo-oligomer (56), had two obvious peaks, one with a M_app_ of ∼380 kDa, and a second peak at ∼80 kDa (Fig. 4*A*). Gaussian fitting was also used to distinguish an unresolved peak in the void from those within the size resolution of the column. For example, The ATPase DNA REPAIR protein had a peak in the void and a second peak that corresponded to a protein with an apparent mass of ∼570 kDa (Fig. 4*B*). Only the resolved peaks are reported in this article, and all proteins that were detected only in the void in the Superdex and superpose columns are flagged as such in supplemental Table 1.

Gaussian fitting was accurate because ∼95% of the fitted peaks fell within one fraction of the global max, and the rare disagreement between the two methods was caused by aberrant maximum data points in the profile. Gaussian fitting enabled the identification of reproducible peaks that had ≤ 2-fraction shift between biological replicates. ∼1500 proteins were resolved as a single peak, ∼150 proteins had one peak in the void and one resolved peak, ∼260 proteins had two reproducible peaks, and only 45 proteins had three or four reproducible peaks (Fig. 4*C*, Gaussian peaks: supplemental Table S1). Proteins with multiple peaks have multiple reported apparent masses and are indicated with the prefix P1, P2, or P3 in decreasing apparent mass (supplemental Table 1). In cases where the data did not allow Gaussian fitting but was reproducible, the global max was used to measure M_app_.

##### Validation of the SEC Profiling Method

**Fig. 5.**
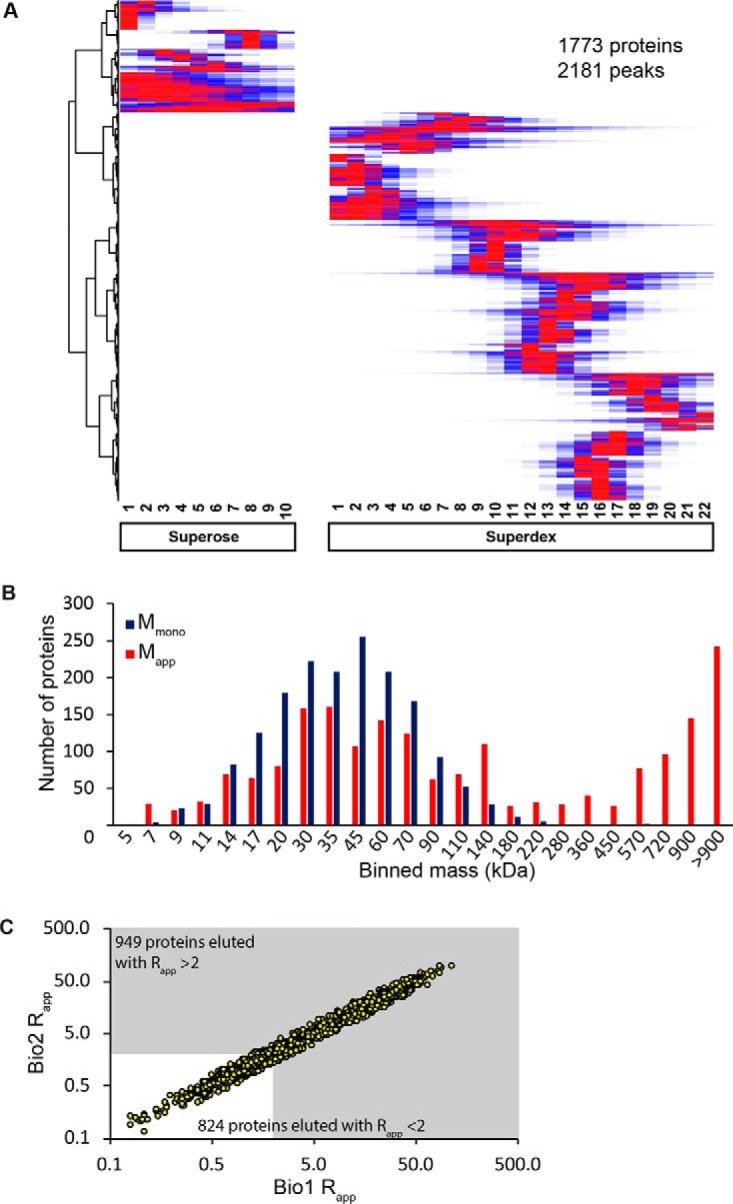
**A high proportion of microsomal proteins are likely to exist as a stable complex.** *A*, Hierarchical clustering analysis was performed based on the reproducible deconvoluted Gaussian fitted peaks for each protein normalized 0 to 1 in Bio1. Red represents the fractions of peak intensity and blue represents fractions were the protein was identified at a lesser intensity. *B*, Evidence for widespread oligomerization. For the proteins detected in this study, the distribution of the measured apparent masses (red) was compared with the calculated monomeric masses (blue). *C*, A scatter plot of the R_app_ values for all of the proteins in the two biological replicates that had reproducible peaks. The gray area indicates the 949 proteins that are predicted to elute as a complex based on an R_app_ threshold of 2 or greater.

We observed strong evidence for the oligomerization of microsomal proteins. A hierarchical clustering analysis was performed on the fitted profiles of ∼2180 peaks belonging to ∼1770 proteins by the similarity of their elution profiles (Fig. 5*A*, Supplemental Searchable analysis Searchable hierarchical clustering analysis). The clustering analysis showed hundreds of proteins coeluting in the same fraction with peaks across the entire resolving range of the column. The histogram of the apparent mass values suggest many proteins are likely oligomeric because of the obvious skew away from the proteome average monomeric mass of ∼50 kDa (Fig. 5*B*).

We used the R_app_ ratio as a metric to predict oligomerization. When the R_app_ is ≥2 in one biological replicate, the protein is predicted to form a complex because its four-fraction shift from the M_mono_ of a protein, making it unlikely that the elution shift is because of chance (29). We do not claim that this represents a homodimer, only that its migration on the column is consistent with protein complex formation. This somewhat arbitrary threshold will generate many false negatives. Some false positives are expected because of elongated shapes of monomeric proteins and extensive protein glycosylation that cause aberrant mobilities on an SEC column. However, we found no systematic increase in R_app_ values for proteins that are glycosylated or acylated (supplemental Fig. S2*A*). The calculated R_app_ values were not influenced by protein abundance, because R_app_ was not correlated with protein signal intensity (supplemental Fig. S2*B*). M_mono_ has the potential to influence R_app_ for proteins at the extremes of the size distribution, but overall, there was no correlation between R_app_ and the calculated mass of the monomer (supplemental Fig. S2*C*). Monomeric mass did not strongly influence the oligomerization predictions (supplemental Fig. S2*D*), but some small proteins had a propensity for a much larger R_app_. We found that many hydrophobic proteins containing one or more transmembrane domains eluted as complexes (supplemental Fig. S2*E*); however, there was not a large difference between the percent of zero, one, or multiple transmembrane domain containing proteins that were predicted with an R_app_ ≥2 (supplemental Fig. S2*F*). We did not expect cholate to nonspecifically influence protein oligomerization because a similar detergent solubilization and SEC analysis protocol with antibodies had been validated using mutants (36, 57). We found no evidence for cholate-based artifacts here, because the apparent mass values of soluble proteins previously reported (29) were nearly identical to those reported here (supplemental Fig. S2*G*). Therefore, the apparent masses measured here appear to be reliable. A scatter plot of the R_app_ values for all of the proteins with reproducible elution profiles in the two biological replicates is shown in Fig. 5*C*. Of the ∼1770 proteins that were analyzed, ∼950 had a R_app_ ≥2 and were predicted as oligomeric. Many of the complexes were quite large, and ∼600 proteins had a R_app_ ≥10.

**Fig. 6.**
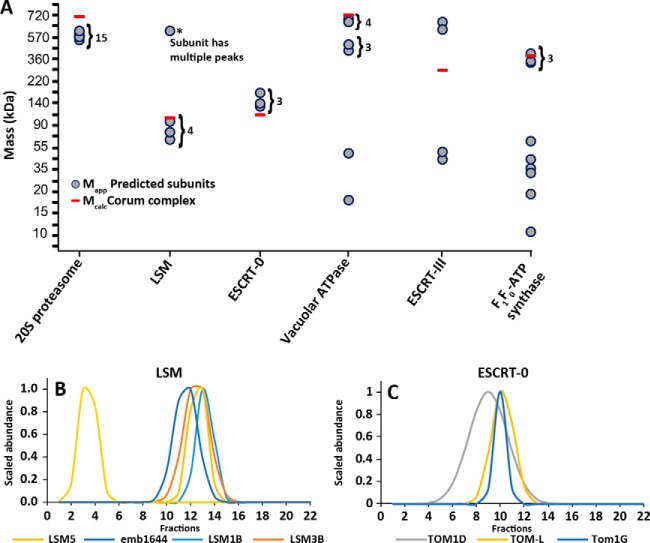
**Most conserved membrane-associated complexes are partially assembled.** *A*, Arabidopsis orthologs to known metazoan protein complexes that are archived in the Corum database were identified and the deduced mass of the conserved fully assembled complex (red bars) was compared with the measured apparent masses (gray circles) of the subunits that correspond to the given metazoan complex. *B*, The elution profile of 4 subunits from the LSM complex. *C*, The elution profiles of the 3 subunits of the ESCRT-0 complex.

Known protein complexes can serve as a useful resource to validate profiling based measurements of apparent mass (28, 32). We manually searched our data set for known protein complexes. The uncapped proteasome is an abundant, predominantly cytosolic complex that is a likely contaminant in the washed microsomes. Nonetheless, we reproducibly measured apparent masses for 15 of the 23 known subunits of the core complex, and they all coeluted with an apparent mass of ∼470, which was close to the calculated mass (Fig. 6*A*). The LSM complex required for mRNA processing and turnover (58) was shown to elute with an average R_app_ of ∼6 as predicted because LSM is known to form a heptameric complex. Additionally, one subunit LSM5 contained two peaks; one eluted with the assembled complex and the second with a M_app_ of ∼500 kDa which is much larger than the known complex (Fig. 6*B*). The data tended to be less clear-cut for other known membrane-associated complexes. The V-ATPase proton pump is a heteromeric complex required to generate the proton gradient with a V_1_ sector facing the cytosol, and a V_0_ membrane spanning sector (59). We identified a partially assembled V_1_ complex by four subunits with an average M_app_ of ∼650 kDa and a three subunit V_1_ subcomplex of ∼370 kDa (Fig. 6*A*). Protein binding assays have shown the association between the coeluting V_1_ subunits *in vitro* (59). The endosomal sorting complex required for transport (ESCRT) are sequential protein complexes that distort membranes to form multivesicular bodies to degrade proteins containing membrane-spanning domains (60). ESCRT-0 is required for the selection of ubiquitinated proteins and was identified by three coeluting subunits at a M_app_ of ∼120 kDa that was smaller than the predicted complex (Fig. 6*C*). Escort-III forms large oligomers that drive membrane scission, and it was identified by four subunits: two isoforms of VPS20, SNF7B, and CHMP2A (Fig. 6*C*). The VPS20 subunits that are known to interact with membranes were predicted as a large complex while CHMP2A and SNF7B eluted as monomers (60). Profiling identified 7 of 15 known subunits from the mitochondria F_1_F_0_ ATP synthase. Three of the subunits were shown to elute with the expected mass of ∼290 kDa and belong to the F_1_ catalytic sector, the remaining subunits were identified as small complexes and monomers (Fig. 6*A*).

We next wanted to broadly compare our mass determinations to the mass of fully assembled known protein complexes. Arabidopsis lacks a curated database of known protein complexes like the CORUM database (43) of metazoan complexes. Therefore, we generated a database that matched Arabidopsis orthologs to subunits of known metazoan protein complexes. This is valid as many of these complexes carry out conserved functions in all eukaryotic cells, and there is evidence that many complexes are conserved across kingdoms (30). First, the comprehensive resource of mammalian protein complexes (CORUM) (43) was used as a source of knowns, and Arabidopsis orthologs were identified using inParanoid (45) a sequence-based predictor and MetaPhors a phylogeny-based predictor (44). Over 2100 Arabidopsis orthologs were matched to about half of the proteins in CORUM. We predicted 853 plant complexes with: high coverage (orthologs identified for ≥80% subunits, 3 of 4 subunits, or 2 of 3 subunits); 117 complexes with medium coverage (60% ≤ orthologs ID < 80%); and 72 complexes with low coverage (40% ≤ orthologs ID < 60%) (supplemental Table S2).

In general, the orthologous Arabidopsis and mammalian complexes were very similar in mass based on a scatterplot of their calculated masses (R^2^ = 0.92) (supplemental Fig. S3*A*). Therefore, we could compare the experimentally measured apparent masses of the Arabidopsis orthologs with the calculated mass of the fully assembled mammalian complex. Surprisingly, there was no correlation between the calculated complex mass and the experimentally determined apparent mass (supplemental Fig. S3*B*). In a recent publication, a weak correlation was reported for predicted and measured cytosolic complexes that were analyzed in mammalian cells (30). Certainly, the fully assembled and activated complex is of major functional importance. However, our results suggest that, unlike the proteasome core particle, most known complexes do not exist solely as stable, fully assembled complexes.

##### Identification of True Membrane-associated Proteins

**Fig. 7.**
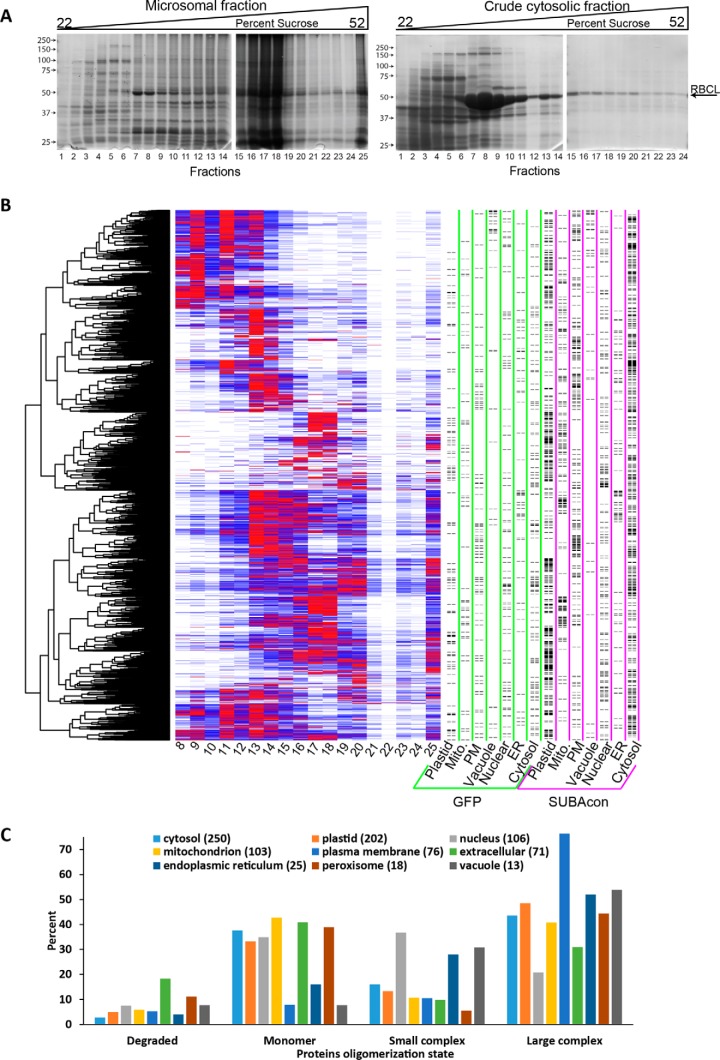
**Identification of true membrane-associated protein complexes and their oligomerization state as a function of subcellular localization.** *A*, Coomassie stained SDS-PAGE gels of the fractions collected from the microsomal (left) or S200 crude cytosolic fraction (right) that were layered and resolved on continuous 22–52% linear sucrose gradients. *B*, The raw XIC intensities for the protein abundance profiles from fractions 8–25 were subjected to hierarchical clustering. The intensity for each protein was normalized from 0–1 based on the max intensity in the replicate. Red lines show fractions of peak intensity with blue lines indicating fractions of lesser intensity. The predicted localization of the protein of interest is marked with a black dash based on GFP data (green columns) or SUBAcon consensus predictions (magenta). *C*, Oligomerization state as a function of known localization. The R_app_ values of membrane-associated proteins were grouped by their SUBAcon subcellular localization. Degraded proteins were defined as those with an R_app_ < 0.5; monomers 0.5≤ R_app_<2; small complexes 2≤ R_app_<10; and large complexes R_app_ ≥10.

Our next goal was to use sucrose velocity gradient separation of microsomes and known marker proteins to identify true membrane-associated proteins and assign a subcellular localization. This has been done using crude microsomes isolated from callus (61). When washed microsomal vesicles (P200) fraction or soluble proteins (S200) were separated on paired identical gradients, their mobilities on the gradient were very different. The vast majority of the proteins in the P200 penetrated deeply into the gradient and reflected the stable association of the protein with a membrane surface (Fig. 7*A*). Most of the proteins in the S200 were retained in the upper eight-fractions even the ∼540 kDa soluble RUBISCO complex (62) that had a peak centered on fraction 8 and bled across much of the gradient (Fig. 7*A*). We next analyzed each fraction collected from the P200 sucrose velocity gradient for relative protein abundance using LC/MS and XIC quantification (supplemental Table S3). We first filtered the profiles to contain only proteins that had a reproducible Gaussian peak in fractions 8–25. Clustering analysis of the raw abundance profiles was performed on fractions 8–25 with the raw abundances normalized from 0 to 1. Known localizations were plotted on the right (Fig. 7*B*). The proteins with known localizations had scattered distributions across the entire gradient and no organelle fell in a single cluster. Therefore, the sucrose velocity gradient profile data was used to distinguish true membrane-associated proteins from cytosolic contaminants that did not penetrate deeply into the gradient.

Proteins were defined as membrane-associated if they were present in the cholate-soluble profiling but not detected in the cytosol (55) or if they were detected in both cholate-soluble and the cytosol but the protein had a peak in the sucrose gradient beyond fraction 8. There was a very minimal level of contamination of fractions beyond fraction 8, because only the largest cytosolic complexes, the 20S proteasome core particle (750 kDa), and the 26S (2000 kDa) capped proteasome had peaks that were centered on fractions 8 and 11 in the sucrose gradient. Only 12 cytosolic proteins had an apparent mass ≥450 kDa, and they were excluded as possible dual localized proteins. The sucrose velocity gradient identified ∼2000 proteins and ∼1400 had a reproducible peak in both SEC and sucrose velocity gradient profiling experiments. These filtering criteria allowed us to remove ∼440 proteins as likely contaminants, and the remainder were defined as true membrane-associated proteins and were flagged as such in supplemental Table S1.

Of the ∼1965 validated membrane-associated proteins in our data set, ∼1365 had a measurable M_app_ using either the Superdex or the Superose columns. Using R_app_ ≥2 as a metric, 818 proteins were predicted to form a complex, and 71% of them were present in relatively large complexes with a R_app_ ≥10. The supplemental Table S4 reports the apparent mass of all validated membrane-associated proteins. Coeluting proteins can be found by searching the clustering output file (supplemental Fig. S5) by locus ID number. We used SUBAcon localization predictions to analyze oligomerization as a function of subcellular localization (Fig. 7*C*). Extracellular proteins were the only group of proteins with the majority having a R_app_ <2, and many of these proteins were degraded perhaps reflecting an increased susceptibility to protease at the elevated pH of our extraction buffer. The plasma membrane had the highest proportion of oligomeric proteins; 87% were predicted to exist in a complex, many of them large.

##### Important Membrane-associated Proteins Identified in Large Complexes

Our profiling method identified proteins that have known importance in plant growth and development. For example, our data set included 126 membrane-associated proteins that have phenotypes (TAIR (01–23-2013)), with some related to lipid trafficking, auxin transport, hypersensitive response and 65% had a R_app_ ≥2 (supplemental Table S1 column J). Plants are sessile organisms, therefore highly integrated signaling networks exist to allow cells to adapt to varying environmental conditions. Many of the networks are controlled by protein kinases (63), and using MapMan (40) and Gene Ontology (64) we identified fifty-five kinases, with 31 of them predicted to be oligomeric (supplemental Table S*1*, column K). Plants contain a large family of sucrose nonfermenting protein kinases (SnRK) that downregulate anabolic pathways under stress conditions to conserve cellular energy (63). SnRK 2 had an elution peak at 330 kDa and an R_app_ of ∼5.

To understand how a protein's oligomerization state connects to its cellular function, we mapped membrane-associated proteins onto pathways by the Kyoto Encyclopedia of Genes and Genomes (KEGG) (65). Coverage in most pathways was sparse, but plant defense had 22 proteins mapped to key nodes (supplemental Fig. S4*A*). Pathogens are sensed by plasma membrane localized receptors such as BRASSINOSTEROID INSENSITIVE 1-ASSOCIATED RECEPTOR KINASE 1 (BAK1) (supplemental Fig. S4*B*) (66). In this study, we identified two BAK1 INTERACTING KINASES, BOTRYTIS-INDUCED KINASE1 (BIK1) (14) and BR-SIGNALING KINASE 1 (BSK1) (67) (supplemental Fig. S4*B*). Each of these interacting proteins had an M_app_ of ∼760 kDa with less than a 2-fraction shift among all three peaks. Plants have proteins that recognize bacterial effector such as RPM1 INTERACTING PROTEIN 4 (RIN4) to activate the hypersensitivity response (68). RIN4 has been shown to physically interact with multiple plasma membrane H^+^-ATPases (AHA-1 and AHA-1) (69). In our sample, we detected AHA-1 and AHA-2 as a single peak in the void; however, AHA-3 and RIN4 had similar elution profiles with an M_app_ of ∼830 and ∼910 kDa, respectively.

##### Many Proteins Are Dual Localized Proteins and Form Distinct Complexes

**Fig. 8.**
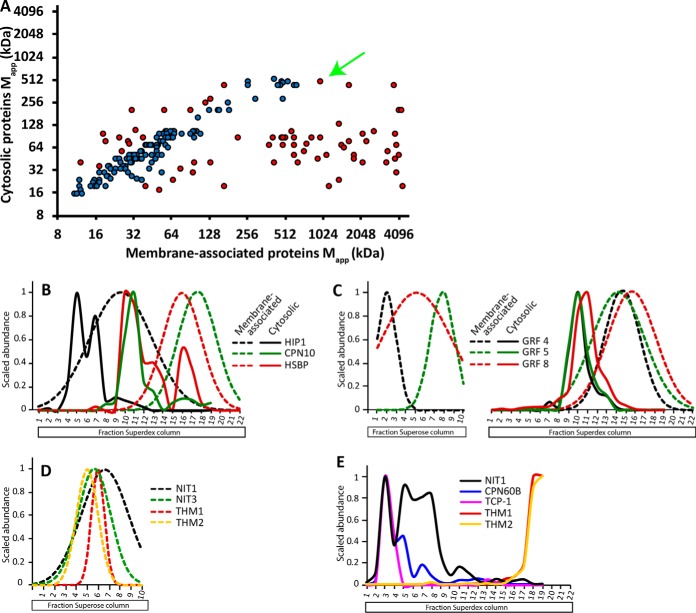
**Many membrane-associated proteins have a dual localization with differing oligomerization states.** *A*, The M_app_ values for proteins that were defined as true membrane-associated and had been previously been analyzed in the soluble fraction independent of cholate solubilization (55). The red points indicate the proteins with a predicted shift in mass between the soluble and membrane fraction by having a ratio of the M_app_ between the cytosol and membrane to be greater than 2 or less than 0.5. The blue points were not predicted to have a shift in mass. The black labeled arrow labels the NITRLASE1 data point. *B*, The abundance profiles of three chaperones coded as black (HIP1), green (CPN10), and red (HSBP) profiles were in larger complexes in the soluble fraction (solid lines) compared with their corresponding membrane-associated form (dashed lines). *C*, Three GRF/14-3-3 isoforms were identified as dual localized. The profiles in the membrane fraction reveal both a large complex with distinct masses and coeluting dimeric forms (dashed lines). The cytosolic fraction contained coeluting isoforms that migrated as trimers (solid lines). *D*, The elution profiles on the Superose column of the membrane-associated proteins that were identified by coIP-MS using the NITRILASE antibody. *E*, Elution profiles of published NITRILASE1 interactors in the soluble fraction from Aryal *et al.*, 2017 (55).

Some proteins localize to multiple subcellular compartments, this is likely a strategy for the cell to control the activity or function of the protein (20). In one scenario, a protein has the same oligomeric state in both compartments, but in a regulatory scheme it is shuttled between the two compartments as active and inactive pools. Alternatively, multiple complexes with distinct oligomerization states may exist to perform different functions. A plot of the apparent masses of the soluble and membrane-associated pools had a pattern in which many of the proteins fell on the diagonal, indicating a similar complex existed at both subcellular locations (Fig. 8*A*). In many instances, the data points fell well off the diagonal and a subset had apparent masses that differed by a factor of two, which is approximately a 4-fraction shift in the peaks. This list included proteins that were in a larger complex in the cytosol. For example, three proteins that function as chaperones and protein folding were in larger complexes in the cytosol (Fig. 8*B*). A greater number of dual localized proteins were in larger complexes in the membrane fraction compared with the cytosol. GRFs function as signaling proteins (70) and multiple isoforms were identified in this study. In the cytosol, all the GRFs coeluted with a R_app_ of ∼3, but the membrane-associated GRFs showed specificity with unique peaks. On the Superdex column, all the GRFs eluted with a R_app_ of ∼2, but the Superose column resolved each GRF as a large complex with a distinct peak (Fig. 8*C*).

To validate the coelution of interacting proteins after SEC separation and test for differing interacting partners in each subcellular locale NITRLASE1 (NIT1), labeled in Fig. 8*A*, was purified by coimmunoprecipitation (CoIP) from the soluble and membrane fractions and MS was used to identify interactors. NITRILASE1 forms a heteroligomer (71) with multiple NIT isoforms, and is important for the detoxification of organic cyanides during pathogen attack (72) and modulating auxin biosynthesis (73). The anti-NIT1 CoIP identified NIT3, and two thioredoxins (THIOREDOXIN M2 (THM2) and THIOREDEXOIN M-TYPE1 (THM1)) as membrane-associated interactors of NIT1. On the Superose column NIT1 coeluted as a large complex containing NIT3, THM1 in both biological replicates and THM2 in only Bio2 (Fig. 8*D*). NIT1 and its putative interactors did not all have a calculated M_app_ in our final table because there was a ∼3 fraction shift in the elution profiles between bio1 and bio2. However, the peak shifts in the biological replicates were similar for the NIT1 and NIT3 isoforms and their predicted interactors, as would be expected for proteins that are in the same complex. In the soluble fraction Aryal *et al.*, 2017 (55) performed coIP-MS using NIT1 antibodies and showed NIT1 interacted with the coeluting proteins CHAPERONIN 60 beta and CHAPERONIN 60 BETA FAMILY PROTEIN (TCP-1). The elution profiles of the soluble NIT1 complex subunits are shown in Fig. 8*E*. Nitrilase appears to have a significant chloroplast-localized pool (25), and likely exists as a soluble complex in the chloroplast lumen stabilized by chaperonins and large chloroplast membrane-associated complexes containing thioredoxins.

## DISCUSSION

Compartmentalization of cellular functions is a defining feature of eukaryotic cells, and membrane-associated protein complexes allow chemical energy, information, and cargo to flow among organelles. In this article, we provide a new protein correlation profiling method to analyze protein complexes across diverse functional categories. Historically, membrane associated proteins have escaped analysis because of the hydrophobic character of the complexes or the lipids with which they interact. Here, we describe a generally useful detergent-solubilization and LC/MS profiling method to analyze the oligomerization state of organelle-associated protein complexes.

Cholate is long known as a useful nondenaturing detergent to solubilize proteins and retain activity (46, 47). Cholate has been used previously in combination with antibodies to solubilize intact membrane-associated protein complexes from leaf microsomes (36, 49). Cholate is effective for the proteomic analyses described here because 70% of the leaf microsomal protein was solubilized, including proteins with a broad range of hydrophobicities (Fig. 2), functionalities (Fig. 3*C*), and subcellular localizations (Fig. 3*B* and 7*C*). Cholate did not have a general tendency to disassemble complexes or promote aggregation, because most dual localized proteins that had both a cytosolic (isolated without cholate (55)) and a membrane-associated pool had very similar apparent masses (Fig. 8*A*). Cholate did not promote aggregation of high abundance proteins, because oligomerization predictions were independent of protein abundance (supplemental Fig. S2*B*). However, solubilization was not perfect; about ∼ 13% of the total number of proteins identified had peaks in the void fractions for both the Superdex (upper mass limit ∼900 kDa) and Superose columns (upper mass limit ∼5 MDa). We report apparent mass values only for those proteins that had one or more reproducible elution peak outside of the void. These data are tabulated in supplemental Table S1, which provides a chromatographic roadmap to native protein complexes of interest for the research community. All raw LC/MS data files are also made available at JPOST repository (74) (PXD006694) (75).

The protein coverage in this study was good, and included hundreds of proteins that had previously escaped analysis, because previous articles analyzed the soluble fraction (Fig. 3*B*). These are proteins with a known importance for plant growth and development, because many have reported mutant phenotypes (supplemental Table S1, column J) or encode proteins with diverse cellular functions (Fig. 3*C*). Our data set included key proteins and large protein complexes containing calcium-dependent protein kinases and the key target of pathogen effectors RIN4 (68) that function in the plant immune response pathway (supplemental Fig. S4*A*). Novel proteins and protein complexes involved in cell wall assembly have been highlighted (supplemental Table S6), because of their widespread importance for plant morphogenesis and the bioenergy sector. Our data set also included proteins that are predicted to localize to all the major organelles (Fig. 7*C*).

Our oligomerization predictions are reliable based on profile data obtained from multiple SEC separations of solubilized membrane-associated proteins. The profile data from our workflow were reproducible because ∼85% of the proteins identified were detected in both biological replicates, and their protein signal intensities across the SEC column fractions in the biological replicates were most highly correlated in identical column fractions (Fig. 3*A* and supplemental Fig. S1*D*). The apparent mass assignments of individual proteins were highly consistent; however, unlike soluble proteins (55), the proteins detected in this study often had profiles with multiple peaks and a global maximum could not be used for peak detection. For example, the LSM5 subunit from the LSM complex had two peaks: one that coeluted with known subunits of the complex, and a second higher mass peak that could reflect a regulatory or second independent function for LSM5 (Fig. 6*B*). Therefore, to account for this behavior we modified a simple Gaussian fitting algorithm (41) to identify instances in which the sizing column could clearly resolve multiple peaks. Gaussian fitting identified ∼2200 resolved peaks mapping to ∼1770 proteins (Fig. 5*A*). Most identified peaks were reproducible, because 70% of the proteins had < 1 fraction peak shift in the two biological replicates, and 85% of the proteins had a peak location within two column fractions, which corresponds to about a ∼40% error in apparent mass. We chose a two-fraction shift as the threshold for reproducibility, and using that metric, we report apparent mass values for ∼1770 proteins that were present in a crude microsome fraction. Of the total, ∼18% had two reproducible peaks and those proteins have unique entries for the same protein in supplemental Table S1.

A common method to validate profiling data is to compare the elution profiles of subunits of known complexes. The proteasome has served this purpose in nearly every protein complex profiling article (28, 29, 32, 55), and here, the proteasome subunits (likely a contaminating cytosolic complex) coelute as expected (Fig. 6*A*). There are relatively few known membrane-associated protein complexes that have been isolated from plant or animal cells, and we did find examples in which a subset of known complex subunits coeluted, probably as partially assembled complexes or protein subunits with multiple functions carried out with distinct binding partners. For example, the V-ATPase subunits identified here have highly variable oligomerization states (Fig. 6*A*). This likely reflects the complex assembly mechanism of the active complex and the distinct subcellular localization patterns that have been published for different V-ATPase subunits (76–78) (Fig. 6*A*).

We also conducted a more global comparison of our experimental M_app_ measurements and the calculated masses of predicted “known” protein complexes. It is important to point out that the “knowns” represent proteins that work together as part of a complex based on genetic and biochemical (often binary interactions detected *in vitro*) interactions, and only in rare cases has the assembly status of the complex been analyzed *in vivo*. By plotting the calculated mass of the fully assembled evolutionarily conserved complexes and the experimentally determined apparent masses, we found no global correlation. A similar analysis conducted with protein complexes from a human cell line also failed to detect a strong correlation between the masses of “known” and experimentally observed protein complexes (30). In plant cells, the assembly status of the known exocyst complex has been analyzed biochemically, and the individual subunits exist in widely varying oligomerization states (79). Perhaps this is a common occurrence as our data indicate that the vast majority of protein complexes do not exist in a stable, fully assembled state that is amenable to biochemical purification. It may be that the fully assembled complexes are often unstable or of low abundance compared with the sub-complexes, and that cellular control of the pools of monomers, partially, and fully assembled complexes has a general regulatory importance. Such a scheme would also allow proteins to carry out multiple independent functions. In any case, there may be relatively few “known” or “golden standard” protein complexes that can be used to validate profiling experiments. Because experimental determination of protein complex assembly *in vivo* is technically challenging, it is not presently known if the predominance of partially assembled complexes is an artifact of cell disruption, which is inherent to any profiling experiment. Therefore, we are reporting on the oligomerization status of individual proteins and not on the intactness of any complex.

In our proteomic pipeline, oligomerization is widespread: ∼50% of the membrane-associated proteins are predicted to exist as some sort of protein complex. This value is higher than the value of ∼30% that was previously reported for cytosolic proteins (55). Our oligomerization predictions are based on the ratio of the measured apparent mass of the protein divided by the predicted mass of the monomer. This is a reasonable method to predict oligomerization (27, 29), and would correspond to roughly a four-fraction shift in the profile peak if the result were because of experimental error alone. The R_app_ values were not driven by protein abundance (supplemental Fig. S2*C*) and the ratio was not strongly affected by the predicted mass of the monomers (supplemental Fig. S2*D*). However, the R_app_ diagnostic has limitations. For example, when M_mono_ is small and the protein binds to a much larger protein (∼4× larger), only the smaller protein will experience a mass shift that allows the complex to be discovered. Further, the mass calculated by SEC is overestimated as proteins shape deviates from globular. We find that proteins with an increased number of TMDs tend to have greater R_app_ values (supplemental Fig. S2*E*). This may reflect either an asymmetric shape of the solubilized protein or true oligomerization. Bulky post-translational modifications such as extensive glycosylation or lipid acylation could inflate R_app_ independent of protein-protein interaction. For example, the fasciclins (FLA)/AGPs can be heavily glycosylated (80). Of the 6 fasciclins that we detected, 4 had extremely high R_app_ values of >40. Glycosylation of fasciclin may not explain the mobility of these proteins on the SEC column because two other FLA proteins (FLA8 and FLA1) had R_app_ values of ∼3–6.

We used the SUBAcon database to analyze oligomerization state as a function of subcellular localization. We found that most cellular compartments had a similar proportion of proteins distributed among small and large protein complexes. However, the plasma membrane was enriched in large protein complexes compared with the other organelles. This may reflect the ability of the plasma membrane to sense and respond to hundreds of endogenous and environmental cues. For example, the plasma-membrane coreceptor BAK1-INTERACTING RECEPTOR-LIKE KINASE 1 (BIR1) that is involved in sterol hormone signaling and plant immunity was in the void (81). However, the downstream signaling components BSK1 and BAK1 coeluted and were detected in large complexes with an M_app_ of ∼800 kDa (supplemental Fig. S4*B*). The plasma membrane localized transmembrane kinase 1 (TMK1) has been implicated in cell morphogenesis, and it had an M_app_ of ∼4 MDa (82). The plants cell wall serves as a protective barrier, controls the patterns of growth, and is a major source of renewable fuel (83). Our proteomics analysis identified dozens of protein complexes that are either cell wall-localized or are involved in cell wall synthesis (supplemental Table S6). Pectin methylesterases (PME) are secreted proteins that are critical for growth control in a variety of cell types and species. PME removes methyl-esters from pectin and allows the polysaccharide to form calcium cross-linked gels that can either promote or restrict cell expansion (84, 85). We detected Arabidopsis PME3 in a ∼1650 kDa complex, pointing to the existence of binding partners because in PME purified from carrot migrates with its expected mass expected on a SDS-PAGE gel and was crystalized as a monomer (86) (supplemental Table S6). We also found six cell wall proteases and glycosyl hyrdolases in the GTX and GTY families in large complexes that may influence cell wall turnover and/or the release of signaling peptides (87).

The profile data from the sucrose velocity gradient allowed to identify true membrane-associated proteins and discover a subset of dual localized proteins that had another detectable protein pool in the cytosol (Fig. 8*A*, supplemental Table S5). As expected, most dual localized proteins had similar M_app_ values, but a few differed considerably at the two cellular locales, suggesting a biological importance. For example, the membrane associated GRFs eluted with two peaks, one consistent with a dimer, and a second that corresponded to a large membrane-associated complex that differed among the isoforms (Fig. 8*C*). The GRFs can be recruited to the membranes to regulate the activity of proton pumps (88), and the distinct peaks for the GRFs may represent stable physical associations with distinct target proteins. In the cytosol, all GRFs coelute with an R_app_ of ∼3, suggesting the presence of mixed homo- or hetero-trimers in the cytosol. Our data are consistent with a model, which a cytosolic pool of inactive trimers are disassembled on membrane surfaces, where individual GRFs interact with specific target proteins. Our validation work on NIT1 further shows that PCP is a valid approach to identify proteins that assemble into distinct protein complexes at different sub-cellular localizations (Fig. 8*D* and 8*E*). A complete list of dual localized proteins and their apparent masses at each location is provided in supplemental Table S5.

In conclusion, we provide a robust new method for a global functional analysis of membrane-associated proteins, a protein class that is only recently yielding to high throughput analysis (35). With our detergent solubilization methods, it is now possible to analyze the native cytosolic and membrane-bound proteins from the same sample, greatly increasing the proteome coverage. Profiling green tissue is particularly challenging because highly abundant chloroplasts dominate the protein samples. SEC-based profiling of subsets of sucrose velocity gradient fractions would lead to further coverage gains. This protein localization and oligomerization analysis pipeline could immediately be applied to analyze protein dynamics as a function of a differing environmental conditions, genotypes, or developmental stages. The sizing column alone does not provide sufficient resolution to accurately predict protein composition, and the challenge of reliably predicting protein complex composition from profiling data alone remains. The combined used of cell fractionation with multiple orthogonal separations and protein correlation profiling is needed to predict the subunits of stable protein complexes.

## DATA AVAILABILITY

The Gaussian fitting code was deposited (https://github.com/dlchenstat/Gaussian-fitting), the raw data and MaxQuant search outputs were deposited at JPOST (PXD006694), and spectra for single peptide hits were deposited at MS-Viewer (89) (http://msviewer.ucsf.edu/prospector/cgi-bin/msform.cgi?form=msviewer) (Search keys: uerfksr5dl, sceybigrnm, osakjoppm2).

## Supplementary Material

Supplemental Data

## Footnotes

^1^ The abbreviations used are:

XIC: Extracted ion chromatogram
BIC: Bayesian information criterion
CORUM: Comprehensive resource of mammalian protein complexes
CoIP-MS: Coimmunoprecipitation-Mass spectrometry
KEGG: Kyoto encyclopedia of gene and genomes
M_app_: Apparent mass measured by size exclusion chromatography
M_calc_: Calculated mass of a predicted protein complex
M_mono_: Calculated monomer mass for a protein
P200: 200,000 × *g* pellet containing the microsomal fraction
R_app_: Ratio of the calculated mass to the apparent mass
S200: Soluble proteins after differential centrifugation
SEC: Size exclusion chromatography
SUBAcon: Arabidopsis consensus subcellular localization
TAIR: The Arabidopsis information resource
TMD: Transmembrane domain.
