# Supplementary material for: Global Analysis of Membrane-associated Protein Oligomerization Using Protein Correlation Profiling
Source: Mol Cell Proteomics. 2017 Sep 8;16(11):1972–89. doi: 10.1074/mcp.RA117.000276 (PMC5672003; doi:10.1074/mcp.RA117.000276)
Supplement: Supplemental Data [file supp_16_11_1972__index.html]

Global analysis of membrane-associated protein oligomerization using protein correlation profiling — Oligomerization predictions for membrane-associated proteins — Global Analysis of Membrane-associated Protein Oligomerization Using Protein Correlation Profiling — Oligomerization Predictions for Membrane-associated Proteins — Supplemental Data 

# Global Analysis of Membrane-associated Protein Oligomerization Using Protein Correlation Profiling

## Supplemental Data

- Supplemental Figures - PDF containing all the supplemental figures
- Supplemental Table S1 - Peak locations, oligomerization state, and raw abundance profiles for proteins identified by SEC profiling.
- Supplemental Table S2 - Complete database of Arabidopsis orthologs to known metazoan complexes and second sheet with the Mapp for proteins identified by profiling.
- Supplemental Table S3 - Peak locations and raw abundance profiles for proteins identified on the sucrose velocity gradient.
- Supplemental Table S4 - A list of proteins predicted to form an oligomeric membrane-associated complex
- Supplemental Table S5 - Oligomierzation state of the dual localized proteins.
- Supplemental Table S6 - Subset of proteins that were predicted to be associated with processes or localized to the cell wall by Mapman, Gene Ontology, and Proteomic studies.
- Supplemental Table S7 - Intensity values and unfiltered data from Nitrilase1 CoIP-MS experiments
