# Supplementary material for: Global Analysis of Membrane-associated Protein Oligomerization Using Protein Correlation Profiling
Source: Mol Cell Proteomics. 2017 Sep 8;16(11):1972–89. doi: 10.1074/mcp.RA117.000276 (PMC5672003; doi:10.1074/mcp.RA117.000276)
Supplement: Supplemental Data [file supp_RA117.000276_4895_0_supp_4973_rrsr0c.pdf]

Figure S1: Microsomal profiling reproducibly characterizes a new set of proteins
(A) The SEC A280 traces for bio 1 and bio2 using a Superdex or Superose column.
(B) Silver stained gel of the 24 Superdex SEC fractions from bio 1 loaded by equal proportions.
(C) A Venn diagram between bio 1 and bio 2 of the peptides and proteins identified by MS/MS across all fractions of the Superdex SEC column.
(D) A cross-correlation comparison of the person correlation coefficient of Bio1 vs Bio2 of the high mass fraction analyzed from the Superose SEC column.
(E) The percent of proteins that contain a transmembrane domain in the two profiling studies of cytosolic proteins, this study, and the proteomic abundance.

Figure S2: Oligomerization predictions are not artifacts due to protein abundance or monomer mass, but proteins with multiple membrane-spanning domains have large R_{app}.
(A) Scatterplot with the R_{app} on the x-axis and protein intensities on the y-axis.
(B) The binned R_{app} of proteins with known and predicted glycosylation and lipid post translation modifications from Uniprot.
(C) Scatterplot of the monomer mass of each protein as a function of $R_{app.}$
(D) To test if a proteins monomer masses skewed complex predictions proteins were binned by M_{mono} and the percent of proteins eluting as a complex ($R_{app}\geq 2$; blue) or monomer/degraded ($R_{app}<2$; green) were plotted.
(E) Scatterplot showing the oligomerization of proteins identified with zero (blue), one (orange), or multiple transmembrane domains (black).
(F) The percent of proteins eluting as a putative complex ($R_{app} \geq 2$) with zero, one, or multiple transmembrane domains.

Figure S3: There is no global correlation between the computationally predicted masses of conserved protein complexes and experimentally determined masses of subunits of conserved protein complexes.
(A) The calculated mass values of the metazoan complexes can be used to compare with the experimental data because the calculated masses of the individual subunits that are present in conserved heteromeric protein complexes are very similar. Calculated metazoan (y-axis) and Arabidopsis (x-axis) subunits were plotted in instances in which there orthologous subunits were detected for all subunits of the complex.
(B) The calculated mass of the predicted fully assembled metazoan complex (y-axis) and the experimentally determined M_{app} of the orthologous Arabidopsis subunit of the complex that were detected in our study (x-axis). Arabidopsis complexes were included in this analysis if medium or high coverage of the subunits were present between the human and Arabidopsis proteomes (see methods).

Figure S4. Oligomerization state of proteins involved in plant immunity.
(A) The KEGG Plant: Pathogen interaction pathway is shown. The membrane-associated proteins in the pathway that were detected here are boxed in red and soluble proteins identified in the S200 profiling from Aryal et al., (submitted) are boxed in green. The R_{app} values of the corresponding protein groups is indicated above each colored box.

(B) The Superdex SEC elution profiles of the BIK1, BAK1, and BSK1 kinases downstream of the BIR1 receptor.

Figure S5: A PDF of the clustering analysis that is searchable by protein locus for the Superdex, Superose, and Sucrose velocity gradient protein correlation profiling experiments.
(A) Clustering analysis of the Supredex fitted profiles
(B) Clustering analysis of the Superose fitted profiles
(C) Clustering analysis on the raw data for fractions 8-25 of the sucrose gradient of the membrane-containing region
(D) Clustering analysis on the raw data for all fractions of the sucrose gradient

Table Legends:

Supplemental Table 1: Peak locations, oligomerization state, and raw abundance profiles for proteins identified by SEC profiling.

Supplemental Table 2: Complete database of Arabidopsis orthologs to known metazoan complexes and second sheet with the M_{app} for proteins identified by profiling.

Supplemental Table 3: Peak locations and raw abundance profiles for proteins identified on the sucrose velocity gradient.

Supplemental Table 4: A list of proteins predicted to form an oligomeric membrane-associated complex

Supplemental Table 5: Oligomierzation state of the dual localized proteins.

Supplemental Table 6: Subset of proteins that were predicted to be associated with processes or localized to the cell wall by Mapman, Gene Ontology, and Proteomic studies.

Supplemental Table 7: Intensity values and unfiltered data from Nitrilase1 CoIP-MS experiments

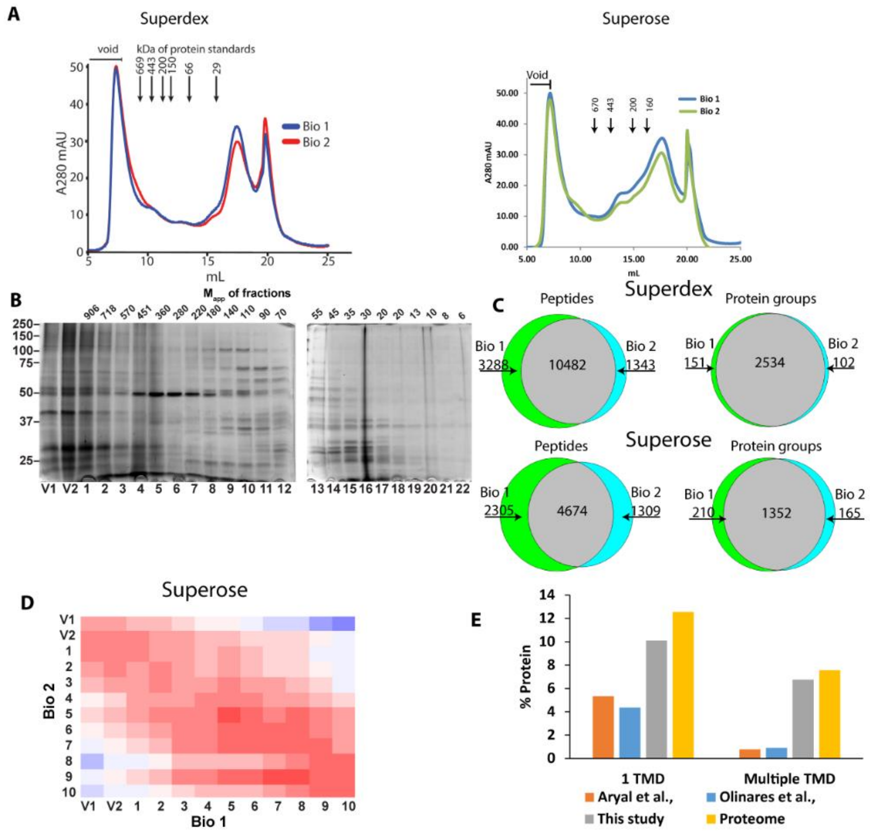
A
Superdex
Superose
void
kDa of protein standards
Bio 1
Bio 2
A280 mAU
mL
Void
B
Mapp of fractions
C
Peptides
Protein groups
Bio 1
Bio 2
10482
2534
4674
1352
D
Superose
Bio 2
Bio 1
E
% Protein
1 TMD
Multiple TMD
Aryal et al.,
Olinares et al.,
This study
Proteome

Figure S1

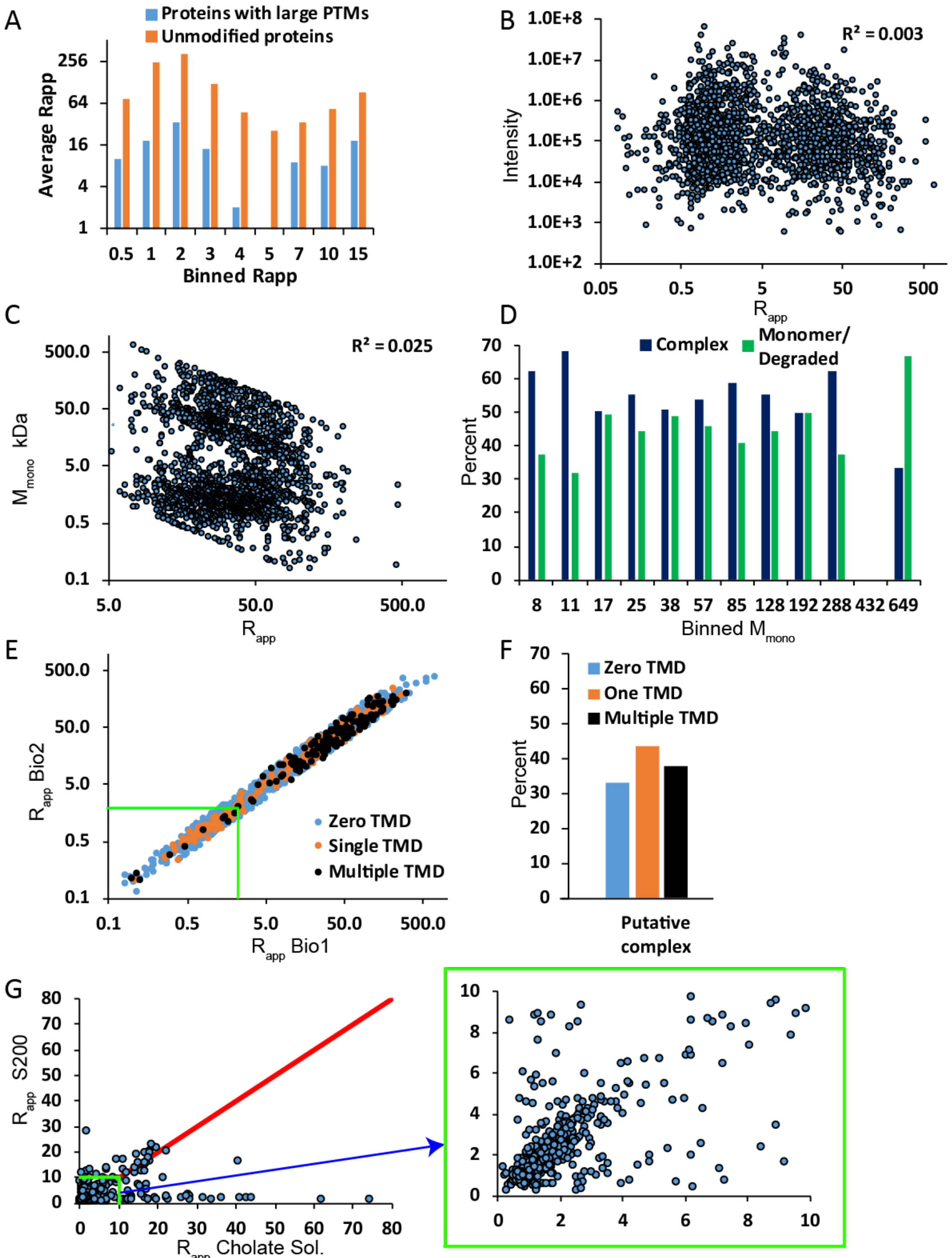
A
Proteins with large PTMs
Unmodified proteins
Average Rapp
256
64
16
4
1
0.5 1 2 3 4 5 7 10 15
Binned Rapp
B
R² = 0.003
Intensity
1.0E+8
1.0E+7
1.0E+6
1.0E+5
1.0E+4
1.0E+3
1.0E+2
0.05 0.5 5 50 500
Rapp
C
R² = 0.025
Mmono kDa
500.0
50.0
5.0
0.5
0.1
5.0 50.0 500.0
Rapp
D
Complex
Monomer/ Degraded
Percent
70 60 50 40 30 20 10 0
8 11 17 25 38 57 85 128 192 288 432 649
Binned Mmono
E
Rapp Bio2
500.0 50.0 5.0 0.5 0.1
Zero TMD
Single TMD
Multiple TMD
0.1 0.5 5.0 50.0 500.0
Rapp Bio1
F
Zero TMD
One TMD
Multiple TMD
Percent
70 60 50 40 30 20 10 0
Putative complex
G
Rapp S200
80 70 60 50 40 30 20 10 0
0 10 20 30 40 50 60 70 80
Rapp Cholate Sol.
10 8 6 4 2 0
0 2 4 6 8 10

Figure S2

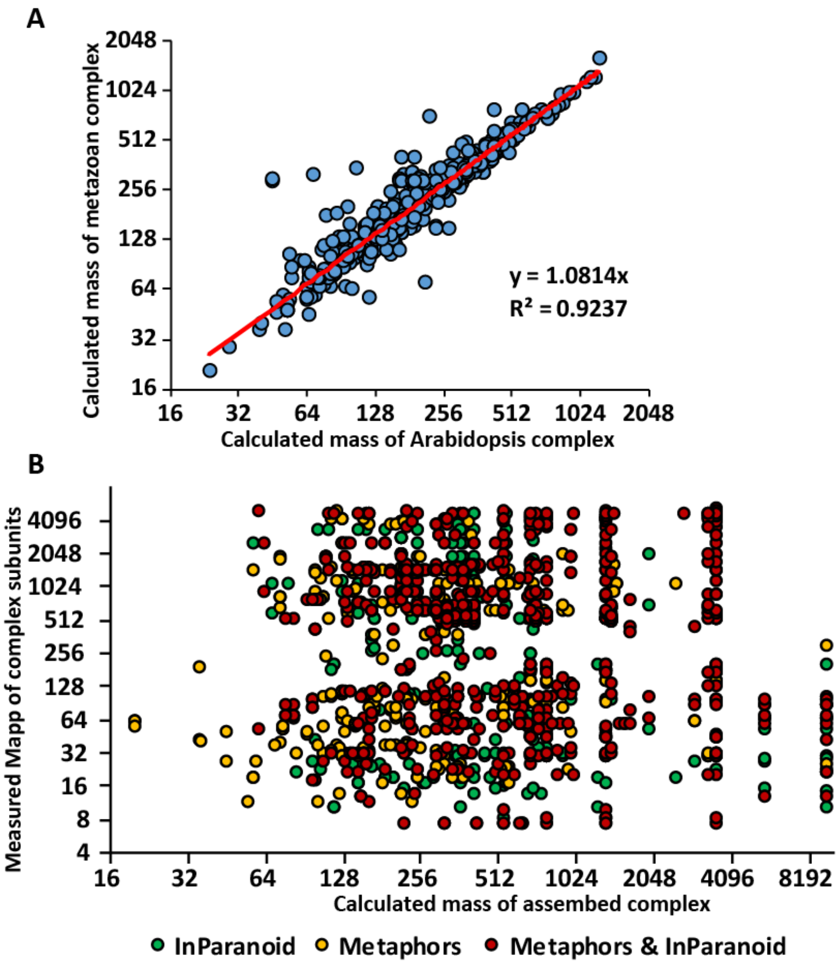
A
2048
1024
512
256
128
64
32
16
Calculated mass of metazoan complex
16
32
64
128
256
512
1024
2048
Calculated mass of Arabidopsis complex
y = 1.0814x
R² = 0.9237
B
4096
2048
1024
512
256
128
64
32
16
8
4
Measured Mapp of complex subunits
16
32
64
128
256
512
1024
2048
4096
8192
Calculated mass of assembed complex
InParanoid
Metaphors
Metaphors & InParanoid

Figure S3

A)

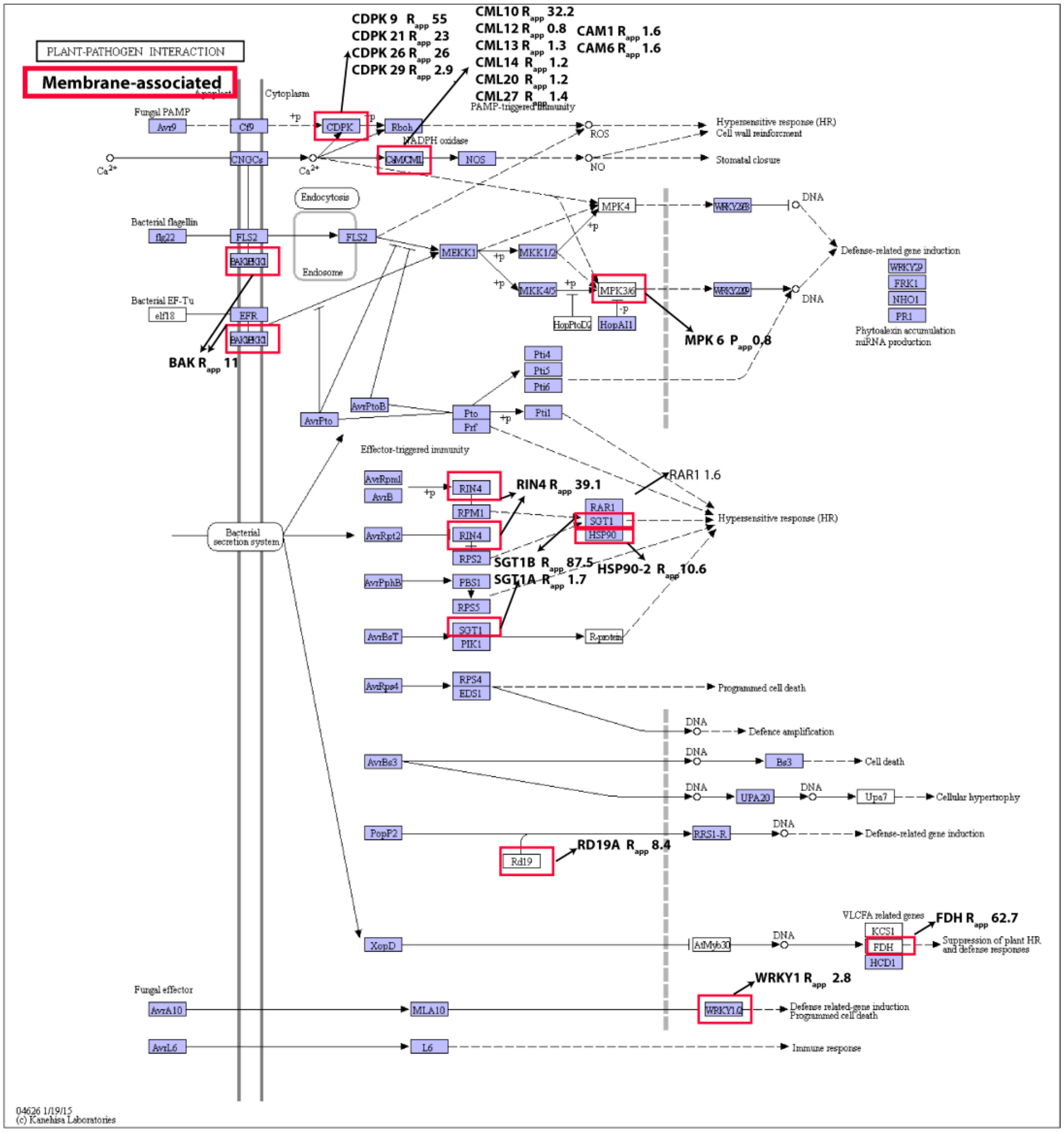
PLANT-PATHOGEN INTERACTION
Membrane-associated
Cytoplasm
Fungal PAMP
Avr9
Cf9
CDPK
Rboh
CNGCs
CaM/CML
NOS
ROS
NO
Hypersensitive response (HR)
Cell wall reinforcement
Stomatal closure
NADPH oxidase
Endocytosis
Endosome
Bacterial flagellin
flg22
FLS2
BAK1/BKK1
MEKK1
MKK1/2
MKK4/5
MPK4
MPK3/6
WRKY25/33
WRKY22/29
DNA
Defense-related gene induction
WRKY29
FRK1
NHO1
PR1
Phytoalexin accumulation
miRNA production
Bacterial EF-Tu
elf18
EFR
HopPtoD2
HopAI1
Pti4
Pti5
Pti6
Pti1
Pto
Prf
AvrPtoB
AvrPto
Effector-triggered immunity
AvrRpm1
AvrB
RIN4
RPM1
AvrRpt2
RPS2
RAR1
SGT1
HSP90
Bacterial secretion system
AvrPphB
PBS1
RPS5
AvrBsT
PIK1
R-protein
AvrRps4
RPS4
EDS1
Programmed cell death
Defence amplification
AvrBs3
Bs3
Cell death
UPA20
Upa7
Cellular hypertrophy
PopP2
RRS1-R
Rd19
Defense-related gene induction
XopD
AtMyb30
VLCFA related genes
KCS1
FDH
HCD1
Suppression of plant HR and defense responses
Fungal effector
AvrA10
MLA10
WRKY1/2
Defense related-gene induction
Programmed cell death
AvrL6
L6
Immune response
PAMP-triggered immunity
CDPK 9 R app 55
CDPK 21 R app 23
CDPK 26 R app 26
CDPK 29 R app 2.9
CML10 R app 32.2
CML12 R app 0.8
CML13 R app 1.3
CML14 R app 1.2
CML20 R app 1.2
CML27 R app 1.4
CAM1 R app 1.6
CAM6 R app 1.6
MPK 6 P app 0,8
BAK R app 11
RAR1 1.6
RIN4 R app 39.1
SGT1B R app 87.5
SGT1A R app 1.7
HSP90-2 R app 10.6
RD19A R app 8.4
FDH R app 62.7
WRKY1 R app 2.8
04626 1/19/15
(c) Kanehisa Laboratories

B)

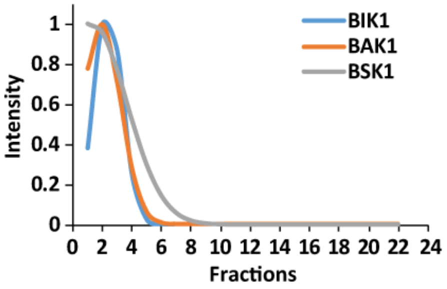
BIK1
BAK1
BSK1
Intensity
1
0.8
0.6
0.4
0.2
0
0 2 4 6 8 10 12 14 16 18 20 22 24
Fractions

Figure S4

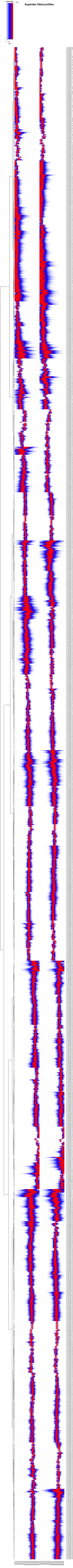
S5A
Superdex fitted profiles
Color Key
0 0.4 1
Value

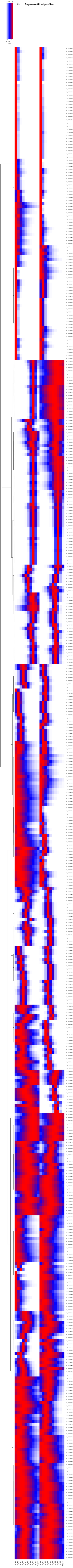
Color Key
0
0.4
1
Value
S5B
Superose fitted profiles

S5C

Sucrose gradient membrane region

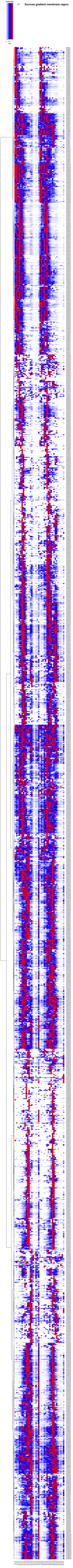

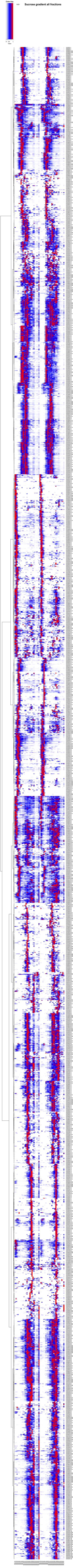
Color Key
0
0.4
1
Value
S5D
Sucrose gradient all fractions
